# The Efficacy of Adjuvant Hyperbaric Oxygen Therapy in Chronic Wound Management: A Narrative Review

**DOI:** 10.7759/cureus.92728

**Published:** 2025-09-19

**Authors:** Sneha Sahay, Sung Yeon Kwak

**Affiliations:** 1 Internal Medicine, University of Bristol, Bristol, GBR; 2 Medical Education, Great Western Hospital, Swindon, GBR

**Keywords:** chronic non healing wounds, diabetic foot ulcers (dfus), hyperbaric oxygen therapy (hbot), venous leg ulcers, wound care management, wound healing

## Abstract

Chronic wounds, or wounds which progress unfavourably through normal stages of healing, place a heavy burden on individuals, impeding their quality of life, and on healthcare systems, with their management annually costing the United Kingdom National Health Service over £5.6 billion. Hyperbaric oxygen therapy (HBOT) has been evidenced for use in a variety of ailments including burns and dive-related injuries. Its physiological effects, such as the stimulation of angiogenesis, bactericidal and bacteriostatic activity, and enhancement of antibiotic potency, have inspired research into its use in the management of hard-to-heal wounds.

This narrative review aims to synthesize recent literature to conclude whether adjuvant HBOT improves the healing of chronic wounds when compared with traditional treatment methods. This information could be used to determine whether HBOT should be made a more mainstream treatment option for chronic wounds.

A literature search and screening process resulted in 11 studies, which were reviewed, encompassing a variety of chronic wound types. The primary outcome was the rate of wound healing, in terms of complete healing and/or reduction in wound surface area. Secondary outcomes included quality of life, pain, amputation rates, improvement of underlying conditions, and bacterial burden of the wounds.

There were some disparities in the literature; while some studies found HBOT to drastically improve the outcome(s), others found no statistically significant difference in healing between the HBOT and standard treatment groups. However, these disparities may be attributable to limitations in the studies or review design.

Certainly, there was no evidence that HBOT worsened outcomes compared to traditional management; it was therefore recommended that it be considered earlier as an option for treatment-refractory wounds. When HBOT is unavailable or deemed inappropriate, it is recommended that other methods of oxygen delivery into the wound, such as oxygen dressings, be considered, to avail at least some of oxygen’s physiological benefits.

## Introduction and background

Chronic wounds, defined as wounds which progress through the physiological stages of healing in an abnormal or untimely fashion [1], affect more than 3.8 million patients in the United Kingdom alone, costing the National Health Service over £5.6 billion annually [2]. The high burden placed by these wounds on individuals and healthcare systems alike has motivated a plethora of research into ways to ameliorate the healing process. Hyperbaric oxygen therapy (HBOT), or the administration of 100% oxygen in a chamber at pressures higher than atmospheric (>1 ATA), has been investigated historically for its use in diabetic foot ulcers (DFUs), and more recently for other extremity wounds such as venous leg ulcers (VLUs), ischaemic ulcers secondary to thromboangiitis obliterans (TAO), and treatment-refractory surgical wounds [3].

Mechanism of HBOT in wound healing

Some of the main factors contributing to wound chronicity are poor circulation (leading to tissue hypoxia), venous insufficiency, and underlying infections [4]. Administering 100% oxygen at a pressure of 3 ATA creates a plasma dissolved-oxygen concentration equal to the natural concentration achieved by haemoglobin-bound oxygen [5], thus in a person with normal haemoglobin levels, HBOT doubles the oxygen concentration in the bloodstream. This hyperoxygenation increases the oxygen concentration gradient between the blood and tissues, stimulating angiogenesis, which allows for oxygen and vital nutrients to reach the wound and waste products to be excreted more efficiently, reducing inflammation. Furthermore, increased oxygen results in increased reactive free radicals, which are toxic to bacterial cells. Many antibiotics, such as fluoroquinolones and aminoglycosides, rely on oxygen for transport across cell membranes. Therefore, hyperoxygenation also plays a role in increasing antibiotic potency.

Aim

This narrative review aims to synthesize the research from the last 15 years to determine whether HBOT, when used alongside standard of care (SOC) treatment, improves the healing process of chronic wounds (CWs) compared to the SOC alone.

## Review

Methods

**Figure 1:**
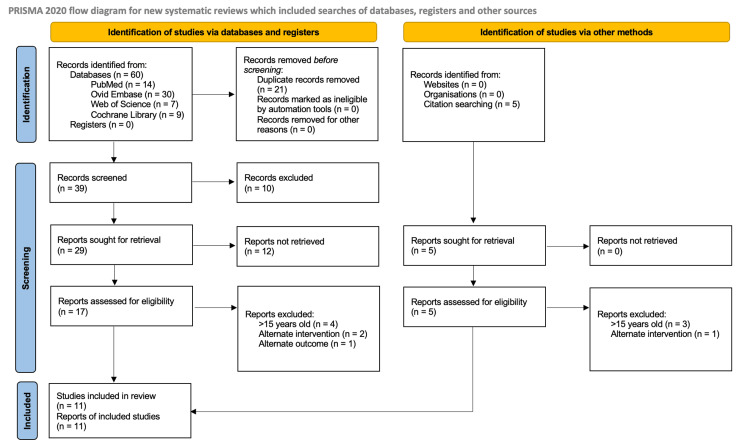
PRISMA Flow Chart for Article Inclusion/Exclusion PRISMA: Preferred Reporting Items for Systematic Reviews and Meta-Analyses [6]

A set of Boolean search terms was generated for the research question (see below). These terms were pasted into four databases: PubMed, Embase, Web of Science, and Cochrane. The result was 60 abstracts, which were exported to Rayaan AI, which identified 21 duplicates, resulting in 39 remaining studies. Through manual Preferred Reporting Items for Systematic reviews and Meta-Analyses (PRISMA) guided abstract and full-text screening, 29 articles were excluded or could not be retrieved, leaving 10 papers to be included in the review [6]. Additional articles were identified through citation searching of the included papers; after repeating the screening process on these, one paper was added to the final review. Therefore, 11 studies were included in the review (see Figure 1). Criteria for exclusion included articles that were over 15 years old or that had interventions and/or outcomes that did not align with the research question. No risk of bias assessment was conducted.

Boolean Search Terms

("chronic wound*" OR "non-healing wound*" OR "diabetic ulcer*" OR "pressure ulcer*" OR "venous ulcer*" OR "leg ulcer*" OR "ischemic wound*") AND ("hyperbaric oxygen therapy" OR "HBOT") AND ("standard treatment" OR "traditional treatment" OR "conventional therapy" OR "usual care") AND ("wound healing" OR "tissue repair" OR "healing rate" OR "treatment outcome" OR "clinical improvement").

The data was heterogeneous with variations in the interventions, outcomes, study designs, and populations. Therefore, a meta-analysis was unable to be performed. A narrative review was undertaken, with each study being assessed for the overall trend in the results rather than specific data values as these were not always comparable.

Results

The findings of the 11 included studies have been summarized in Table 1.

**Table 1:** Summary of Included Studies HBOT: Hyperbaric oxygen therapy; PPS: Persistent perineal sinus; TAO: Thromboangiitis obliterans; VAS: Visual analogue scale; ABPI: Ankle-brachial pressure index; DFU: Diabetic food ulcer; CRP: C-reactive protein; SOC: Standard of care; VLU: Venous leg ulcer

Authors and Year	Aim	Study Type	Type(s) of wound	Outcome(s) being assessed	Summary of findings	Limitations
Chan et al., 2014 [7]	“To explore perioperative HBOT with rectus abdominis myocutaneous (RAM) flap repair in a highly selected group of patients with extreme PPS who had failed all other interventions.”	Case series	Persistent perineal sinus (post-proctectomy chronic surgical wound)	Time to complete wound healing, further hospital admissions required	4 patients studied. All 4 had complete wound healing within 3 months and none needed further hospital admission within the time frame of the study.	No control group. Delivery of HBOT pre- and post-op varied (number of sessions) between patients, follow-up time post-HBOT also varied. Small number of patients (4).
Hemsinli et al., 2016 [8]	“To investigate the effects of HBOT used in addition to classic treatment in [patients with TAO].”	Single-arm study	Ischaemic ulcers as a result of thromboangiitis obliterans	Fontaine wound classification, pain score, walking distance without pain, ability to perform routine activities, wound area	After HBOT, 52.6% of patients recovered fully. There was also a significant decrease in VAS scores for pain, wound area, and Fontaine grade for those who didn’t fully recover, and an increase in ability to walk and partake in daily activities.	Small number of patients (36). Lack of other objective quantitative outcomes, e.g., transcutaneous oximetry and ABPI. No control group in this study; however, a retrospective audit was done 2 years later (Hemsinli et al., 2018) which compared these outcomes to previous TAO patients who had not received HBOT.
Hemsinli et al., 2018 [9]	“To investigate the effect of hyperbaric oxygen treatment (HBOT) in patients with TAO.”	Retrospective audit	Ischaemic ulcers as a result of thromboangiitis obliterans	Major amputation rate, healing of ischaemic wounds, pain score, level of healing, change in Rutherford grade (all after 10 months)	After 10 months, the number of major amputations, as well as VAS pain score and median Rutherford grade, was reduced in the HBOT group. There was no significant difference in wound area or duration of hospitalization between the two groups. There was significantly more complete healing after 10 months in the HBOT group.	Control group was retrospective, so not necessarily under the same conditions as the HBOT group. Short (10 months) follow-up. Low sample size due to the rarity of TAO.
Kaplan et al., 2017 [10]	“To investigate the efficacy of HBOT in the management of DFUs and identify amputation predictors.”	Single-arm study	Diabetic foot ulcers	Complete healing, partial healing, non-healing, or amputation at 3 months, 6 months, 12 months, and then every 6 months for at least 3 years or until amputation/death	Of 146 patients, 87.5% had wound improvement over the course of the study (69.6% complete healing, 17.9% partial healing). 23.2% of cases resulted in amputation. Among others, major amputation predictors were found to be Wagner grade, CRP, longer duration of diabetes, and formation of new wounds.	No control group. No objective quantitative outcomes like transcutaneous oximetry or ABPI.
Kumar et al., 2020 [11]	“To evaluate the efficacy of hyperbaric oxygen therapy (HBOT) as an adjuvant to standard therapy for treatment of diabetic foot ulcers.”	Double-blind randomized controlled trial	Diabetic foot ulcers	Number of cases with: complete healing (no surgical intervention), requiring surgical intervention/debridement, requiring amputation, no change in condition – followed up weekly for 6 weeks, then at 3-month intervals for a year	HBOT was found to have 8x more positive outcomes compared to the control group. No ulcers in the control group healed without surgical intervention compared to 78% in the HBOT group. Healing time was significantly lower in the HBOT group as was the number of proximal and distal amputations.	Transcutaneous oximetry was carried out on admission but not during follow-up, and measurements were not taken at the site of the ulcer. Short follow-up period (1 year). Sample size of n=30 per group with some dropouts, so ultimately n=28 and n=26 in the treatment and control groups, respectively.
Longobardi et al., 2020 [12]	“To ascertain whether the simultaneous measurement of hemoglobin O2 saturation (StO2) and dimension of venous leg ulcers (VLU) by near infrared spectroscopy (NIRS) imaging can predict the healing course with protocols employing a conventional treatment alone or in combination with hyperbaric oxygen therapy (HBOT).”	Randomized controlled trial	Venous leg ulcers	Wound size, transcutaneous oximetry and haemoglobin oxygen saturation, and assessment of healing course over time measured using NIRS 2D imaging and another fluorescence imaging device	There was no significant difference in TcPO2 values of any group at the beginning and end of the study. After 3 weeks, wound area reduction was not significantly different in any of the groups. However, at the end of the study, the less aggressive HBOT regimen and the control group showed a larger decrease in wound area compared to the aggressive regimen. Tissue oxygen saturation decreased over the course of the study for wounds which healed, but did not change in the non-healing wounds.	Short duration of the study which prevented further data collection on the difference in outcomes between various HBOT regimens. Single-center study.
Patil et al., 2024 [13]	“To evaluate whether hyperbaric oxygen can decrease major amputation rates and achieve wound healing in chronic non-healing wounds.”	Prospective observational study	Foot wounds of various pathologies in diabetic patients: peripheral arterial disease, vasculitis, venous ulcer, necrotizing fasciitis, burns	Number of cases with complete healing, requiring surgical intervention, requiring amputation. Transcutaneous oximetry values before and after HBOT	65% of the patient group underwent complete healing after the HBOT sessions. 17% required further surgical intervention and 21% eventually required amputation. Some poor prognostic markers were identified including the wounds being Wagner grade 4, increasing age of the patient and duration of the wound. TcPO2 measurement prior to the HBOT sessions was not found to be an accurate prognostic marker when used on its own.	No control group (no group not receiving HBOT). There was a wide variety in the demographics of the patients and the etiology of their wounds which makes it difficult to determine how useful HBOT was and whether there were other factors influencing wound healing. There was no conclusion drawn which would help to identify which patients in particular would benefit from HBOT.
Salama et al., 2019 [14]	“To assess the efficacy of systemic HBOT in healing of chronic nonischemic diabetic foot ulcer.”	Randomized controlled trial	Non-ischaemic diabetic foot ulcers	Achievement of complete closure of the wound, rate of ulcer healing (measured by surface area) at week 0, 4 and 8 following treatment, and rates of amputation	Complete wound closure rate at week 0, 4 and 8 was 33.3%, 46.7%, and 66.7% in the treatment group and 0%, 13.3%, and 20% in the control group, showing a significant difference. A similar trend was seen in the average wound surface area at weeks 0, 4 and 8. There was no significant difference in the amputation rates between groups – there was 1 minor amputation in each group.	Small sample size – 15 patients per group.
Sanford et al., 2018 [15]	“To examine the efficacy of hyperbaric oxygen therapy (HBOT) towards reducing or eliminating bacterial biofilms in vitro and in vivo.”	In-vitro study and retrospective cohort study	All chronic wounds	Cell viability of bacterial biofilms in vitro when treated with HBOT for 30, 60 and 90 minutes. Bacterial burden/load for patients with chronic wounds who had received a SOC package and an SOC+HBOT package before and after the treatment period (retrospectively)	In vitro, the HBOT group consistently had slightly lower cell viability compared to the control group which suggests some level of bactericidal activity by HBOT, although these results were only statistically significant at the 30 and 90-minute points. In vivo, both groups had a reduced bacterial load after the treatment period, but the results were not statistically significant. In the HBOT group, there was a marked reduction in the spread of data which may suggest more reduction in bacterial activity on average compared to the control (SOC) group.	The in vitro aspect of the study could not account for human immune system involvement and also was not tested with the regular standard-of-care package (which includes antibiotics, etc.) while these were used in the in vivo part of the study. The in vivo part was retrospective and so subject to various confounding factors such as patient age, comorbidities, and the severity of the wounds. Thus, this study makes it difficult to draw conclusions or understand relationships between the in vitro and in vivo results.
Shukla et al., 2020 [16]	“To evaluate the efficacy of HBOT in the management of diabetic ulcer using the Bates-Jensen Wound Assessment Tool.”	Randomized controlled trial	Diabetic foot ulcers	Change in Bates-Jensen wound tool score over the treatment period, length of hospital stay, number of amputations required	Using the Bates-Jensen wound tool to assess wound severity, it was found that in the HBOT group, there was a significantly higher decrease in score between the 0^th^ and 20^th^ sessions compared to the control (SOC) group in the same time frame. There was no significant difference between the 20^th^ and 30^th^ sessions. The HBOT group also had a significantly lower average hospital stay length compared to the control (33.68 days vs 58.44 days). Amputation rate was 4% in the HBOT group and 24% in the SOC group.	Sample size of 25 patients per group.
Thistlethwaite et al., 2018 [17]	“To determine the effectiveness of Hyperbaric Oxygen Therapy (HBOT) as an adjunct treatment for nonhealing venous leg ulcers.”	Double-blind randomized controlled trial	Venous leg ulcers	Number of wounds with complete healing after 12 weeks. Pressure ulcer scale for healing (PUSH) score, reduction in wound area, and pain severity score after 12 weeks	There was no significant difference in the number of people achieving complete wound healing after 12 weeks between the 2 groups. The HBOT group had a significantly higher percentage surface area reduction compared to the control group (95% vs 54%). Changes in PUSH score (to assess wound severity), as well as pain scores and quality of life scores, were not significantly different between the groups.	Small sample size in the intervention phase didn’t allow for randomization of confounding factors; as a result, the HBOT group had patients with worse prognostic factors (age, wound size, etc.) to begin with. The short follow-up time of 12 weeks didn’t allow for complete healing of all the wounds, so may have prevented some trends from being identified.

Wound Healing

The primary outcome of this review was wound healing, which can be measured as the number of wounds undergoing complete healing as well as the rate of clinical improvement of the wound over time.

Complete healing: Time to complete wound healing, or the number of wounds undergoing complete healing, was an outcome in almost all the studies being reviewed. The uncontrolled studies suggested that administration of HBOT contributed to a higher rate of complete healing; Chan et al. followed patients with extreme treatment-refractory surgical wounds, all of whom recovered fully after pre- and post-operative HBOT [7]. Similarly, the patients in Hemsinli et al. were refractory to standard treatment, and 52.6% of them recovered fully after HBOT [8]. A similar trend is seen in Kaplan et al. and Patil et al. [10,13]. The controlled studies had a more mixed outcome - while Hemsinli et al., Kumar et al., and Salama et al. found improved rates of complete healing in the HBOT groups compared to the controls [9,11,14], Thistlethwaite et al. [17] found no significant difference. Longobardi et al. observed a higher incidence of complete healing in a low-intensity treatment group but not a higher-intensity one when compared to the control [12].

Wound surface area: When wounds did not close completely, their healing was measured by their percent area reduction (PAR) over time. Longobardi et al. used near-infrared spectroscopy to monitor the change in wound surface area (WSA) between an intensive HBOT regime, a lower-intensity regime, and a control group. They found that while there was an absolute PAR in all the groups, it was proportionally higher in the low-intensity group and the control group compared to the high-intensity group (61.6% and 52.4% compared to 25.1%) [12]. Similarly, Hemsinli et al. found no significant difference in WSA after treatment between the HBOT and control groups [9]. Conversely, Salama et al. saw a significant PAR (73.3%) immediately post-treatment in the HBOT group, which was not seen in the control group (6.25%). This trend continued at follow-up, with the treatment group PAR reaching 100% while the control group remained at 56.25% after eight weeks [14]. Thistlethwaite et al. saw similar results with the mean PAR being 95% in the HBOT group and 54% with the placebo [17].

Bates-Jensen wound assessment tool: Shukla et al. [16] used a specific tool to assess DFU severity after 0, 10, 20 and 30 HBOT sessions. In the HBOT group, the mean score was 55.16 pre-treatment with 96% of wounds being classified as ‘extreme’. After 30 sessions, the mean score was 20.58 with 100% of wounds improving to ‘moderate’. Comparatively, in the control group over the same time frame, the mean score only improved from 54.12 to 41.42, and while 100% of wounds were classified as ‘extreme’ pre-treatment, 57.9% retained this classification at the end of the treatment period.

Pressure Ulcer Scale for Healing (PUSH) score: Thistlethwaite et al. used the PUSH score to determine the severity of VLUs over time. They found the score decreased over the treatment period in both the HBOT and the placebo group, with no significant difference between the two (p=0.051) [17].

Secondary Outcomes

The reviewed literature comprised various secondary outcomes, which may indirectly reflect HBOT’s efficacy in a wound healing context.

Bacterial burden: Sanford et al. [15] found evidence that HBOT contributed to wound healing by retrospectively comparing the data of CW patients who had received HBOT to those who hadn’t. The HBOT group was found to have a lower healing time (5.8 vs 6.6 weeks) and a greater reduction in bacterial burden, suggesting a relationship between these variables. However, this data was not statistically significant (p=0.06).

Pain: Hemsinli et al. used the visual analogue scale (VAS) pain score in their 2016 and 2018 papers, asking patients to rate their pain from 1-10. The 2016 paper found a significant decrease in the mean VAS score (7.1 to 2.2) after HBOT delivery [8]; the 2018 audit found that at 10 months post-treatment, the HBOT group had a mean score of 1 while the SOC group had a mean score of 6 [9]. Contrarily, Thistlethwaite et al. found no significant difference in pain score improvement between the HBOT and placebo groups [17].

Quality of life and activity level: Hemsinli et al. found that the proportion of patients who were able to complete their routine activities independently increased from 25% to 55.5% after HBOT delivery [8]. They also found the average pain-free walking distance increased from 0 m to 190.6 m after treatment. However, Thistlethwaite et al. found no significant difference in the change in quality of life (QoL) scores between the HBOT and control groups when measured using the SF-12v2 Health Survey [17].

Hospital stay/Re-admission: Chan et al. found that no patients required re-admission after receiving HBOT despite numerous prior admissions [7]. Kumar et al. [11] found 100% of their control group required surgical debridement of their wounds, and thus a longer hospital stay, to facilitate healing, while 78% of the HBOT group healed without any further intervention; Shukla et al. [16] had similar findings.

Amputation rate: While Kumar et al. [11] and Shukla et al. [16] found the amputation rate to be significantly higher in the control group compared to the HBOT group, Salama et al. [14] found an equal number of amputations in each group. Kaplan et al. and Patil et al. aimed to identify amputation predictors in foot wounds; some of these were high Wagner grade, increasing patient age, wound duration, and duration of comorbidities like diabetes [10,13].

Symptom grading: The Fontaine and Rutherford grading scales were used by Hemsinli et al. in 2016 and 2018, respectively, to measure the symptoms of the underlying TAO rather than the ulcer severity. The 2016 study, in which all patients were Fontaine class IV to begin with, saw a significant number of improvements to class IIB following HBOT [8]. Similarly, the 2018 audit found that the HBOT group had significantly more Rutherford grade I and fewer grade III patients compared to the control [9].

Discussion and limitations

The majority of the research being reviewed indicates some advantage to using HBOT adjuvant to the SOC in CW management. This benefit can likely be attributed to HBOT’s local and systemic physiological effects.

The results between studies varied - while some found almost immediate improvements in the rate of complete wound healing and a faster reduction in WSA, others found no significant difference in these outcomes between HBOT and control groups. Similar disparities were found in secondary outcomes such as pain scores, QoL, and amputation rates. These differences can potentially be attributed to the diversity in the study designs, populations, interventions, and outcome measures:

Study Designs

Only seven of the 11 studies were controlled; of these, the control group was retrospective in two cases. The remaining randomized controlled trials (RCTs) had limitations of their own, including limited sample sizes and flaws in randomization, which created biases. A full summary of the limitations of each paper can be found in Table 1; it is reasonable to accredit some differences in trends to these.

Populations

Nine out of the 11 studies focused on a specific wound type (e.g., DFUs, VLUs). This creates challenges in determining whether their results can be applied to CWs as a whole and limits comparisons between the research. For example, the improvement in wound severity in Shukla et al. [16] with HBOT cannot be accurately compared to the results of Thistlethwaite et al. [17], which showed no significant difference in improvement, as the former was investigating DFUs and the latter VLUs.

Interventions

While HBOT was administered in all the studies, each one used different regimes, administering 100% oxygen with differing pressures, periods of time, and numbers of sessions, even within the same study. Longobardi et al. studied one group receiving two HBOT sessions per day for three weeks compared to another group receiving one session per day for six weeks, and found that the latter group had significantly better outcomes [12]. Given the difference in results within that study, it is reasonable to assume that differences between the studies can be attributed to inconsistencies in the HBOT regimes. Similarly, each study defined the control group slightly differently, likely because the SOC varies between hospitals and different wound types. The control groups all received different levels of care, which hinders inter-study comparisons.

Outcomes

The use of scoring systems as outcomes was useful in understanding the findings of individual papers but hindered comparability between studies. For example, while Thistlethwaite et al. [17] used the PUSH score to monitor ulcer severity, Shukla et al. [16] used the Bates-Jensen Assessment Tool, providing values which may indicate the same thing but are not interchangeable. It is difficult to determine whether differences in trends between these papers are attributable to the different scoring systems or some other factor.

Clinical application

Practically, the number of facilities equipped to administer HBOT does not match the burden that CWs place on healthcare systems; currently, there are only 10 centres in the United Kingdom equipped to provide it [18]. Therefore, it is necessary to determine which patients would most benefit from HBOT. Some studies in this review only included patients who were refractory to other treatments, in whom HBOT was unanimously shown to be beneficial. However, the mixed RCT evidence provides a challenge in the equitable allocation of HBOT services. Patil et al. identified a high Wagner grade to be a poor prognostic marker for foot wounds [13], although the Wagner grading system comes with its own limitations. Feldman-Idov et al. [19] found TcPO2 > 200 mmHg at the wound site under hyperbaric conditions to be an accurate marker on the usefulness of HBOT; Patil et al. did not support this finding but concluded that a low TcPO2 (<40 mmHg) could predict inferior outcomes.

HBOT has a range of contraindications, including but not limited to pneumothoraces, obstructive lung disease, pregnancy, ear disease, and respiratory infections [20]. Limited cost-benefit analysis has been conducted on whether, and which, relative contraindications can be overlooked when the patient stands to benefit from HBOT in a CW context. Even those who aren’t contraindicated stand to suffer from complications of HBOT - in the studies reviewed, these were most commonly ear discomfort, transient barotrauma, headache, or claustrophobia, but more serious complications could include lung collapse or oxygen toxicity [21].

Where HBOT could be beneficial but is contraindicated or unavailable, alternative strategies could be employed. De Smet et al. [22] summarized research around methods of oxygen delivery into wounds to facilitate healing, including HBOT, topical oxygen therapy, normobaric oxygen therapy, and oxygen dressings, finding them all to be beneficial in different ways.

## Conclusions

The narrative review provided strong evidence towards the benefits of adjuvant HBOT in wound healing. In comparison to the SOC, many studies have found added HBOT to improve or at least not impair wound healing; larger, multi-centre, double-blind RCTs are necessary to strengthen these findings. As of now, the most validated patients to benefit from HBOT are those who have been resistant to other treatments, and this group should be considered for earlier HBOT to reduce the healthcare burden of CWs. Future studies should attempt to isolate other features which may identify patients to receive earlier HBOT, and risk-benefit analysis should be undertaken considering HBOT’s relative contraindications and complications. In the absence of HBOT, other methods of oxygen delivery into wounds should be considered to promote healing.
